# Synaptotagmin-7 links fusion-activated Ca^2+^ entry and fusion pore dilation

**DOI:** 10.1242/jcs.153742

**Published:** 2014-12-15

**Authors:** Kathrin Neuland, Neeti Sharma, Manfred Frick

**Affiliations:** Institute of General Physiology, University of Ulm, Albert-Einstein Allee 11, 89081 Ulm, Germany

**Keywords:** FACE, Calcium, Exocytosis, Fusion pore, Lamellar body, Synaptotagmin

## Abstract

Ca^2+^-dependent regulation of fusion pore dilation and closure is a key mechanism determining the output of cellular secretion. We have recently described ‘fusion-activated’ Ca^2+^ entry (FACE) following exocytosis of lamellar bodies in alveolar type II cells. FACE regulates fusion pore expansion and facilitates secretion. However, the mechanisms linking this locally restricted Ca^2+^ signal and fusion pore expansion were still elusive. Here, we demonstrate that synaptotagmin-7 (Syt7) is expressed on lamellar bodies and links FACE and fusion pore dilation. We directly assessed dynamic changes in fusion pore diameters by analysing diffusion of fluorophores across fusion pores. Expressing wild-type Syt7 or a mutant Syt7 with impaired Ca^2+^-binding to the C_2_ domains revealed that binding of Ca^2+^ to the C_2_A domain facilitates FACE-induced pore dilation, probably by inhibiting translocation of complexin-2 to fused vesicles. However, the C_2_A domain hampered Ca^2+^-dependent exocytosis of lamellar bodies. These findings support the hypothesis that Syt7 modulates fusion pore expansion in large secretory organelles and extend our picture that lamellar bodies contain the necessary molecular inventory to facilitate secretion during the exocytic post-fusion phase. Moreover, regulating Syt7 levels on lamellar bodies appears to be essential in order that exocytosis is not impeded during the pre-fusion phase.

## INTRODUCTION

Ca^2+^ is the key element in regulated exocytosis controlling multiple steps during the exocytic pre- and post-fusion stages. We have recently described a ‘fusion-activated’ Ca^2+^-entry (FACE) during the post-fusion stage of lamellar body exocytosis in primary alveolar type II (ATII) cells. FACE occurs through P2X_4_ receptors on the membrane of lamellar bodies upon fusion of lamellar bodies with the plasma membrane and opening of the fusion pore. The resulting rise in Ca^2+^ is restricted in time and space to the onset and site of vesicle fusion. FACE drives expansion of the initial fusion pore and subsequent surfactant release (Miklavc et al., 2011). However, the molecular links between FACE, the localised increase in Ca^2+^ around the fused lamellar body, and fusion pore dilation or expansion were still elusive. It was the aim of this study to identify such molecular links between FACE and fusion pore expansion.

Although the molecular composition of exocytic fusion pores is still elusive, there is general acceptance that their opening and closure are highly regulated to allow fine tuning of vesicle content secretion (Alvarez de Toledo et al., 1993; Breckenridge and Almers, 1987; Chow et al., 1992; Larina et al., 2007; Obermüller et al., 2005; Vardjan et al., 2009). It has been shown that the duration of the fusion pore open state and diameter depend on the type of stimulation (Vardjan et al., 2007) and that the stable fusion pore diameter depends on the diameter of the fused vesicles (Jorgačevski et al., 2010). The key regulator for fusion pore expansion is Ca^2+^ (Fernández-Chacón and Alvarez de Toledo, 1995; Haller et al., 2001; Hartmann and Lindau, 1995; Lynch et al., 2008; Scepek et al., 1998; Wang et al., 2006). A range of additional factors, including synaptotagmins (Fernández-Chacón et al., 2001; Hui et al., 2005; Jaiswal et al., 2004; Lynch et al., 2008; Segovia et al., 2010), myosin II (Bhat and Thorn, 2009), Munc-18 (Jorgačevski et al., 2011), dynamin (Anantharam et al., 2011) and F-actin (Larina et al., 2007) among others (Jackson and Chapman, 2008), have also been suggested as molecular mediators for fusion pore transitions.

Synaptotagmins are by far the best-studied proteins regulating fusion pore expansion (Jackson and Chapman, 2008). Synaptotagmins are secretory vesicle proteins composed of an N-terminal transmembrane domain and two C-terminal C_2_ domains, C_2_A and C_2_B, each of which binds Ca^2+^ (Fernandez et al., 2001; Perin et al., 1990; Segovia et al., 2010; Sutton et al., 1995). Ca^2+^-binding synaptotagmin isoforms have been found to regulate fusion pore expansion in neurons, neuroendocrine and various other secretory cells (Moghadam and Jackson, 2013). Among these, the ubiquitously expressed synaptotagmin-7 (Syt7) acts as a high-affinity Ca^2+^ sensor (Bhalla et al., 2005) and has been implicated in regulation of fusion pore dynamics in exocytosis of large vesicles in non-neuronal cells including insulin-secreting granules of β-pancreatic cells (Gao et al., 2000), large dense-core vesicles in PC12 cells (Zhang et al., 2010) and lysosomes (Jaiswal et al., 2004). However, it is still unclear how Syt7, and synaptotagmins in general, promote fusion pore expansion – whether they sculpt the fusion pore directly or control fusion pore dynamics indirectly. Recent evidence suggests that Syt7 shapes fusion pore dynamics without directly participating in the fusion pore itself and that its C_2_A and C_2_B domains contribute differentially to fusion pore dynamics (Segovia et al., 2010).

Within this study, we provide evidence that Syt7 provides a molecular link between FACE and fusion pore dilation. Specifically, binding of Ca^2+^ to the C_2_A domain of Syt7 antagonises complexin-2 recruitment to fused lamellar bodies and thereby facilitates fusion pore dilation during the exocytic post-fusion phase. In addition, we also demonstrate that Ca^2+^ binding to the C_2_A domain of Syt7 impedes exocytosis of lamellar bodies during the exocytic pre-fusion phase.

## RESULTS

### Synaptotagmin-7 is expressed in isolated ATII cells and localised on the lamellar body membrane

**Fig. 1.**
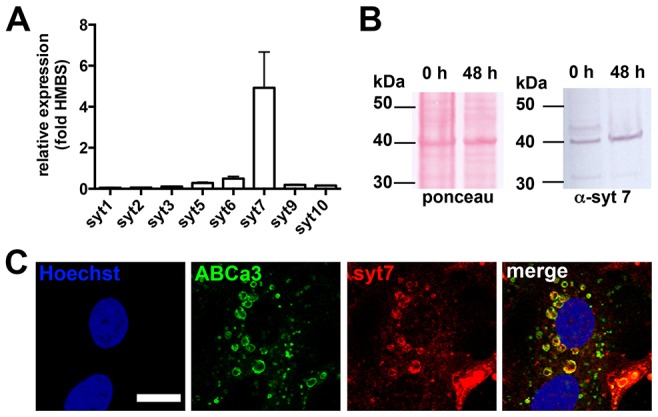
**Syt7 is expressed in isolated ATII cells and localised on the lamellar body membrane.** (A) Real-time RT-PCR analysis of synaptotagmin transcripts in freshly isolated ATII cells from rat. Data are expressed as the relative expression compared to housekeeping gene *Hmbs*. Values are mean±s.e.m. from three individual cell isolations and are represented as mean±s.e.m. (B) Western blot of Syt7 from freshly isolated ATII cells and ATII cells 48 h after isolation confirms expression of Syt7 is not altered in cultured cells. Ponceau staining of blots (left) was used to control for equal loading of lanes. (C) Syt7 (red) is primarily localised on lamellar body membranes as detected by indirect immunofluorescence and confirmed by colocalisation with P180 lamellar body protein (green, ABCa3). Scale bar: 10 µm.

Several molecules have been identified as regulating fusion pore transitions in a Ca^2+^-dependent manner, with synaptotagmins being among the best studied (Jackson and Chapman, 2008). Therefore, we initially investigated the expression of Ca^2+^-sensitive synaptotagmin isoforms in ATII cells using RT-PCR. Our data revealed that Syt7 was by far the most highly expressed isoform in freshly isolated ATII cells (Fig. 1A). Further analysis revealed that Syt7α (280 bp) was by far the most highly expressed splice variant, and there was little expression of Syt7β (410 bp) and almost no expression of Syt7γ (610 bp), in line with previous expression studies in lung tissue (Fukuda et al., 2002). Importantly, expression was not a result of de-differentiation of ATII cells in culture, at least for 48 h after isolation (the maximum period cells were used in functional assays to avoid de-differentiation (i.e. loss of lamellar bodies and cell spreading) and impaired lamellar body exocytosis). Western blotting for Syt7 confirmed that there was no difference in protein levels between freshly isolated cells (30 min after isolation) and cells in culture for 48 h (the maximum time period we use isolated ATII cells for functional studies) (Fig. 1B). Finally, immunofluorescence staining of ATII cells confirmed that Syt7 was primarily localised on lamellar bodies and colocalised with P180 lamellar body protein (ABCa3, Fig. 1C).

**Fig. 2.**
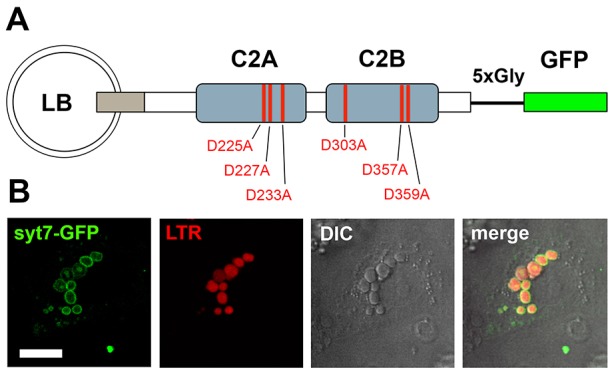
**Syt7–GFP localises to the membrane of lamellar bodies.** (A) Schematic representation of the Syt7–GFP constructs used in this study. Syt7 and GFP are separated by a short glycine linker. Red lines indicate amino acid positions in the Ca^2+^-binding sites in the two C_2_ domains (C_2_A and C_2_B) that were mutated to obtain Syt7 constructs with altered Ca^2+^-binding properties. (B) Syt7–EGFP (green) expressed for 24 h in ATII cells is primarily localised on the limiting membrane of lamellar bodies, as confirmed by co-staining of lamellar bodies with LTR (red). Scale bar: 5 µm.

To test whether Syt7 provides a link between FACE and fusion pore expansion we expressed either mutant or wild-type Syt7 [Syt7(wt)] in primary rat ATII cells. Syt7 was linked to GFP through a short glycine linker, which has been demonstrated to promote proper folding and maintain function of synaptotagmins (Saegusa et al., 2002; Tsuboi and Fukuda, 2007). Syt7 mutants were deficient in Ca^2+^ binding to either the C_2_A [Syt7(C_2_A*)], the C_2_B [Syt7(C_2_B*)] or both C_2_ domains [Syt7(C_2_A*C_2_B*)] (Fig. 2A) (Maximov et al., 2008). Syt7–GFP expressed at endogenous levels was almost exclusively localised on the limiting membrane of lamellar bodies (Fig. 2B), similar to the localisation of endogenous Syt7 (Fig. 1C). ATII cells were analysed, at the latest, at 48 h after isolation to exclude effects of de-differentiation on lamellar body exocytosis.

### Syt7 facilitates fusion pore expansion following exocytic fusion of lamellar bodies with the plasma membrane

**Fig. 3.**
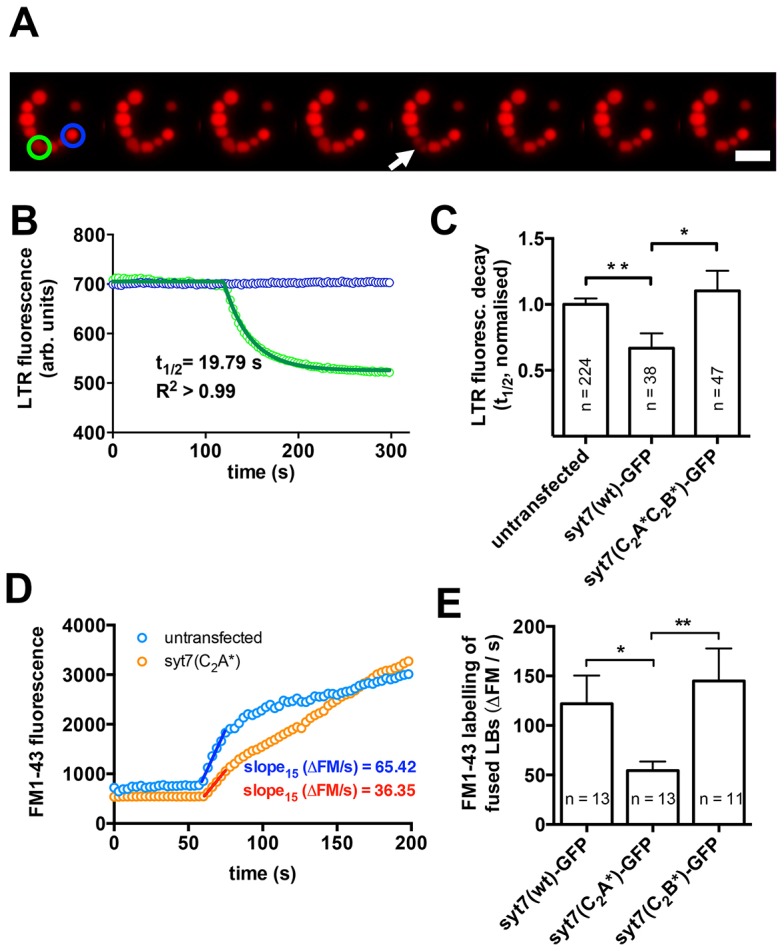
**Syt7 facilitates fusion pore expansion following exocytic fusion of lamellar bodies with the plasma membrane.** (A) Image sequence illustrating loss of LTR from an individual lamellar body following fusion with the plasma membrane and fusion pore opening (green circle). Note that the LTR fluorescence of a non-fusing vesicle (blue circle) does not change significantly. Scale bar: 5 µm. (B) Half-times (*t*_1/2_) of the fluorescence decay were analysed to compare diffusion of LTR across the fusion pore for various experimental conditions and to identify differences in fusion pore opening. To analyse *t*_1/2_ of the fluorescence decrease upon fusion pore opening the fluorescence of fusing vesicles (green circle in A) was normalized to that of non-fusing vesicles (blue circle in A) to compensate for bleaching, and the decrease of fluorescence was fitted to a one-phase decay. (C) Expression of Syt7(wt)–GFP significantly (*P* = 0.006) increases the speed of LTR diffusion from fused vesicles indicating faster fusion pore expansion. However, diffusion of LTR from fused lamellar bodies in cells expressing Syt7–GFP that is deficient in Ca^2+^-binding to the C_2_A and C_2_B domain [Syt7(C_2_A*C_2_B*)–GFP] was not different to wild-type cells and was significantly (*P* = 0.03) slower than in cells expressing Syt7(wt)–GFP. (D) Following lamellar body fusion, FM1-43 fluorescence increases owing to incorporation of the dye into the lipidic vesicle contents (Haller et al., 1998). The initial slope of the FM1-43 fluorescence increase (15 s after fusion) was analysed as a direct measure of FM1-43 diffusion across the fusion pore following lamellar body fusion. (E) Diffusion of FM1-43 into fused lamellar bodies was significantly slower in cells expressing a Syt7 mutant deficient in Ca^2+^-binding to the C_2_A domain [Syt7(C_2_A*)–GFP] when compared to cells expressing Syt7(wt)–GFP or Syt7(C_2_B*)–GFP. Results are mean±s.e.m. *P<0.05; **P<0.01.

To investigate the impact of Syt7 on fusion pore expansion, we next analysed the destaining kinetics of a fluorescent dye that accumulates in intracellular lamellar bodies (LysoTracker Red, LTR) upon fusion of a lamellar body with the plasma membrane and opening of the fusion pore (Fig. 3A,B). A change in fusion pore diameter or expansion rate results in a change in the rate of diffusion of the dye from fused vesicles (Haller et al., 2001; Miklavc et al., 2011). Initial experiments revealed that expression of Syt7(wt)–GFP significantly increased loss of LTR from fused vesicles, with half-times of fluorescence decay being 33.2% lower (*P* = 0.006) than in untransfected cells. However, when a Syt7 mutant deficient in Ca^2+^-binding to the C_2_ domains [Syt7(C_2_A*C_2_B*)–GFP] was expressed no difference compared with wild-type cells was observed and half-times of LTR decay were significantly higher than in cells expressing Syt7(wt)–GFP (*P* = 0.03) (Fig. 3C). These results indicate that Syt7 probably promotes fusion pore expansion in a Ca^2+^-dependent manner in primary ATII cells.

This effect was specific for expression of Syt7 and its high affinity for Ca^2+^. Expressing Syt1 or Syt4, isoforms that have been shown to impact on fusion pore expansion in mammalian cells (Lai et al., 2013; Zhang et al., 2009), but have C_2_ domains with lower Ca^2+^ affinity (Syt1; Geppert et al., 1994) or that do not bind Ca^2+^ (Syt4; Dai et al., 2004), did not affect fusion pore expansion. Syt1(wt)–GFP and Syt4(wt)–GFP also localised on lamellar body membranes, but half-times of LTR fluorescence decay were not significantly altered in cells expressing Syt1(wt)–GFP or Syt4(wt)–GFP when compared to untransfected cells, respectively (supplementary material Fig. S1).

In a complementary assay, we analysed initial diffusion rates of FM1-43 into newly fused lamellar bodies (Miklavc et al., 2011) (Fig. 3D). FM1-43 is a fluorescent dye that is essentially non-fluorescent in aqueous solutions but yields a bright signal when incorporated into lipid layers, the main content of lamellar bodies (i.e. surfactant) (Haller et al., 2001; Haller et al., 1998). Diffusion of FM1-43 into fused lamellar bodies was significantly slower in cells expressing a Syt7 mutant deficient in Ca^2+^-binding to the C_2_A domain [Syt7(C_2_A *)–GFP] when compared to cells expressing Syt7(wt)–GFP (*P* = 0.03) or a Syt7 mutant deficient in Ca^2+^ binding to the C_2_B domain [Syt7(C_2_B*)–GFP, *P* = 0.009] (Fig. 3E). Hence, Ca^2+^-dependent fusion pore expansion through Syt7 is dependent on Ca^2+^ binding to the C_2_A domain of Syt7.

### Ca^2+^ binding to the C_2_A domain of Syt7 facilitates FACE dependent fusion pore expansion

**Fig. 4.**
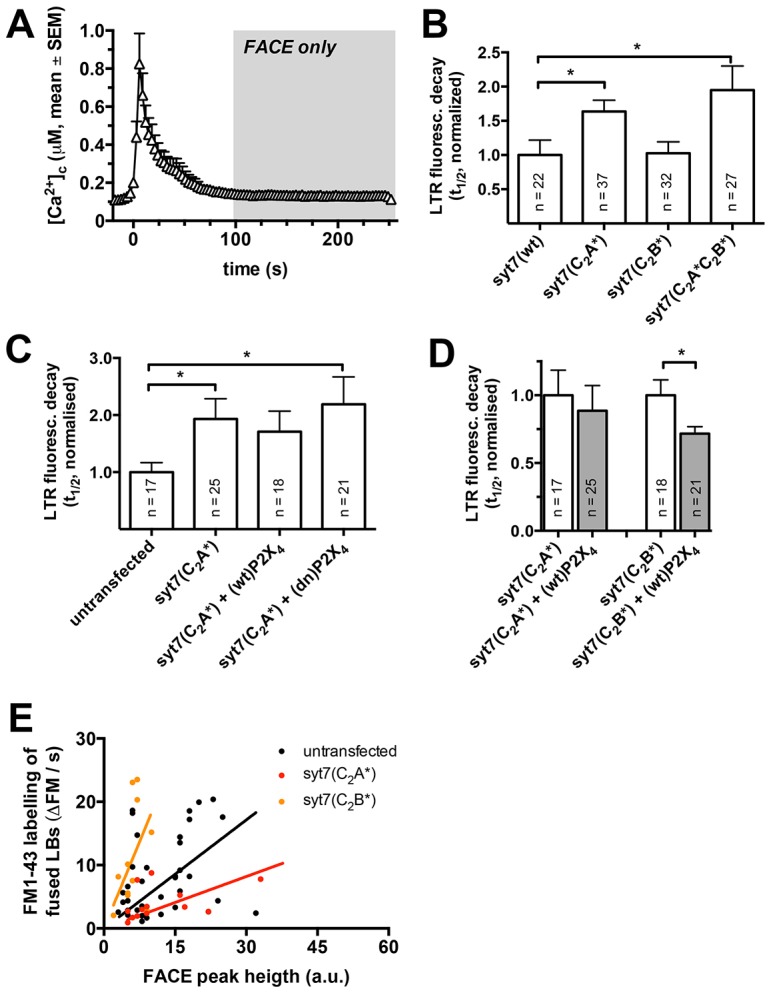
**Ca^2+^ binding to the C_2_A domain of Syt7 facilitates FACE dependent fusion pore expansion and surfactant secretion.** (A) Stimulation of ATII cells with ATP (*t* = 0), results in a transient rise of the intracellular Ca^2+^ concentration. To delineate the impact of FACE from the effect of the global rise in Ca^2+^ on Ca^2+^-dependent effects of Syt7 on fusion pore expansion, only fusions occurring >100 s following stimulation were analysed (when the global Ca^2+^ peak has ceased). *n* = 23 cells. (B) Deletion of Ca^2+^-binding to the C_2_A domain of Syt7 [Syt7(C_2_A*)–GFP or Syt7(C_2_A*C_2_B*)–GFP] significantly increased the halftimes of LTR fluorescence decay when compared to Syt7(wt). Expression of Syt7(C_2_B*)–GFP (intact C_2_A domain) did not affect LTR fluorescence decay when compared to cells expressing Syt7(wt)–GFP. (C) Modulating the amplitude of FACE by overexpressing either wild-type P2X_4_ [(wt)P2X_4_] or (dn)P2X_4_ (Miklavc et al., 2011) did not affect half-times of LTR fluorescence decay in cells expressing Syt7(C_2_A*)–GFP. (D) In contrast to cells expressing Syt7(C_2_A*)–GFP increasing the amplitude of FACE in cells expressing Syt7(C_2_B*)–GFP significantly increased the speed of LTR diffusion from fused lamellar bodies. (E) The rate of FM1-43 diffusion into fused vesicles at given amplitudes of FACE is increased in cells expressing a functional Syt7 C_2_A domain [Syt7(C_2_B*)] and decreased in cells expressing Syt7 with impaired Ca^2+^ binding to its C_2_A domain [Syt7(C_2_A*)] when compared to wild-type cells. Results are mean±s.e.m. *P<0.05.

Based on the findings that Ca^2+^ binding to Syt7, in particular the C_2_A domain of Syt7, facilitates fusion pore expansion, we next wanted to test whether Syt7 links FACE and fusion pore dilation. Stimulation of lamellar body exocytosis in ATII cells with ATP, the prerequisite to also induce FACE through activation of P2X_4_ receptors (Miklavc et al., 2011), results in a transient, initial rise of the intracellular Ca^2+^ concentration (Dietl et al., 2012; Frick et al., 2001; Haller et al., 1999) (Fig. 4A). To delineate the impact of FACE on Ca^2+^-dependent effects of Syt7 for fusion pore expansion from the global rise in Ca^2+^, we only analysed lamellar body fusions occurring at least 100 s following stimulation with 100 µM ATP, when the global Ca^2+^ peak had ceased (Fig. 4A). Again, deletion of Ca^2+^ binding to the C_2_A domain of Syt7 significantly increased the halftimes of LTR fluorescence decay when compared to Syt7(wt). Halftimes were increased by 63.6% (*P* = 0.02) and 94.6% (*P* = 0.03) in cells expressing Syt7(C_2_A*)–GFP and Syt7(C_2_A*C_2_B*)–GFP, respectively, compared to cells expressing Syt7(wt)–GFP. In line with the results above, expression of Syt7(C_2_B*)–GFP, where the C_2_A domain is intact, did not affect LTR fluorescence decay when compared to cells expressing Syt7(wt)–GFP (*P* = 0.92) (Fig. 4B). These results indicate that FACE-dependent fusion pore dilation depends on Ca^2+^ binding to the C_2_A domain of Syt7. Consistently, similar experiments in cells stimulated with either 100 µM UTP or 300 nM PMA, both conditions where FACE is not activated (Miklavc et al., 2011), did not show any difference in LTR fluorescence decay between cells expressing Syt7(wt)–GFP, Syt7(C_2_A*)–GFP and Syt7(C_2_B*)–GFP (supplementary material Fig. S2).

To further investigate the link between FACE, Ca^2+^ binding to Syt7 C_2_A and fusion pore dilation, we performed experiments modulating the size of the local fusion-activated Ca^2+^ signal. It has been shown that the amplitude of FACE is significantly increased in cells overexpressing wild-type P2X_4_, whereas FACE is significantly decreased or totally ceases in cells overexpressing the dominant-negative mutant P2X_4_(C353W) [denoted (dn)P2X_4_] (Miklavc et al., 2011). This modulation of the amplitude of FACE has also been demonstrated to directly impact on fusion pore kinetics, with fluorescent marker diffusion directly correlating to the amplitude of FACE (Miklavc et al., 2011). However, when we modulated the amplitude of FACE by overexpressing either (wt)P2X_4_ or (dn)P2X_4_ no difference in the half-times of LTR fluorescence decay could be observed. Again, expression of Syt7(C_2_A*)–GFP significantly (*P* = 0.04) increased half-times of LTR fluorescence decay (i.e. reduced rate of diffusion of LTR from fused lamellar bodies) compared to wild-type cells. More interestingly, neither an increase [Syt7(C_2_A*) + (wt)P2X_4_] nor a decrease in the amplitude of FACE [Syt7(C_2_A*) + (dn)P2X_4_] significantly affected halftimes of LTR fluorescence decay in cells expressing Syt7(C_2_A*)–GFP (Fig. 4C). However, in contrast to cells expressing Syt7(C_2_A*)–GFP, increasing the amplitude of FACE in cells expressing Syt7(C_2_B*)–GFP significantly (*P* = 0.02) increases the speed of LTR diffusion from fused lamellar bodies (Fig. 4D). This is further supported by our finding that the rate of FM1-43 diffusion into fused vesicles (see also Fig. 3D; Miklavc et al., 2011) at given amplitudes of FACE is increased in cells expressing a functional Syt7 C_2_A domain [Syt7(C_2_B*)] and decreased in cells expressing Syt7 with impaired Ca^2+^ binding to its C_2_A domain [Syt7(C_2_A*)] when compared to wild-type cells (Fig. 4E). In summary, all these data strongly support our hypothesis that Ca^2+^ binding to the C_2_A domain of Syt7 following the FACE-induced local increase of Ca^2+^ around newly fused lamellar bodies facilitates initial fusion pore dilation.

### FACE and Ca^2+^ binding to the C_2_A domain of Syt7 antagonise complexin-2 recruitment to fused lamellar bodies

**Fig. 5.**
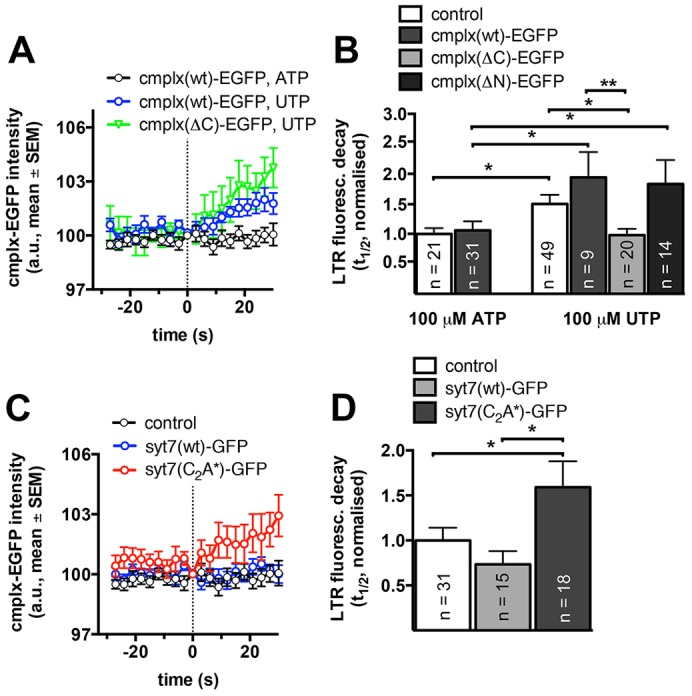
**FACE and Ca^2+^ binding to the C_2_A domain of Syt7 antagonise complexin-2 recruitment to fused lamellar bodies and thereby facilitate fusion pore expansion.** (A) Mean fluorescence of cmplx(wt)–GFP and cmplx(ΔC)–GFP before and after lamellar body fusion with the plasma membrane in cells stimulated with either 100 µM ATP (FACE) or 100 µM UTP (no FACE). FACE inhibits increase of cmplx(wt)–GFP at fused lamellar bodies. Fluorescence change was measured in a peri-vesicular region of interest surrounding individual lamellar bodies. The dotted line indicates the time of fusion (data represent a minimum of ten fusions for each condition). (B) Halftimes of LTR fluorescence decay are not significantly different in cells overexpressing cmplx(wt)–GFP when stimulated with 100 µM ATP (FACE). Stimulation with 100 µM UTP (no FACE) resulted in a significant increase in halftimes of LTR fluorescence decay, which was moderately enhanced in cells overexpressing cmplx(wt)–GFP or cmplx(ΔN)–GFP. In contrast, overexpression of cmplx(ΔC)–GFP completely abolished the increase in the halftime of LTR fluorescence decay following stimulation with 100 µM UTP. (C) Mean fluorescence of cmplx(wt)–GFP before and after lamellar body fusion with the plasma membrane in cells stimulated with 100 µM ATP (FACE). Translocation of cmplx(wt)–GFP to fused lamellar bodies is inhibited in wild-type cells (control, black) and cells expressing Syt7(wt)–GFP (blue) but not in cells expressing Syt7(C_2_A*)–GFP (red). The dotted line indicates the time of fusion (data represent a minimum of ten fusions for each condition). (D) Halftimes of LTR fluorescence decay in cells overexpressing cmplx(wt)-GFP following stimulation with 100 µM ATP. Deletion of Ca^2+^-binding to the C_2_A domain of Syt7 [Syt7(C_2_A*)–GFP] significantly increased the halftimes of LTR fluorescence decay when compared to wild-type cells and cells expressing Syt7(wt)–GFP. Results are mean±s.e.m. *P<0.05.

We next aimed at further elucidating the mechanism of how Ca^2+^ binding to the C_2_A domain of Syt7 following FACE facilitates fusion pore dilation. It has recently been reported that complexin-2 binding to SNARE complexes modulates fusion pore kinetics (An et al., 2010) and that synaptotagmin-1 antagonises complexin-2-mediated restriction of fusion pore expansion in a Ca^2+^-dependent manner in chromaffin cells (Dhara et al., 2014). RT-PCR and immunofluorescence revealed that complexin-2 is expressed in primary ATII cells and that it is predominantly localised in the cytoplasm, respectively (supplementary material Fig. S3). Furthermore, analysing lamellar body fusions occurring at least 100 s following stimulation revealed that EGFP-tagged wild-type complexin-2 [cmplx(wt)–EGFP] increased at the site of lamellar body fusion, indicating cmplx(wt)–EGFP translocation to fused lamellar bodies. This translocation only occurred in the absence of FACE. Upon stimulation with 100 µM UTP but not following stimulation with 100 µM ATP, cmplx(wt)–EGFP fluorescence increased at the site of lamellar body fusion indicating a possible link between FACE and complexin-2 recruitment to fused lamellar bodies (Fig. 5A). Expression of cmplx(wt)–EGFP did not affect FACE-dependent fusion pore expansion (Fig. 5A, 100 µM ATP) and only moderately slowed LTR diffusion from lamellar bodies fused in the absence of FACE (Fig. 5A, 100 µM UTP). However, expression of complexin-2–EGFP lacking the C-terminus [cmplx(ΔC)–EGFP], but not complexin-2–EGFP lacking the N-terminus [cmplx(ΔN)–EGFP], completely abolished the restriction of fusion pore expansion in the absence of FACE (Fig. 5A). Upon stimulation with 100 µM UTP (no FACE), halftimes of LTR fluorescence decay were significantly increased by 49.9% (*P* = 0.04), 83.2% (*P* = 0.01) and 72.7% (*P* = 0.03) in wild-type cells, cells expressing cmplx(wt)–GFP and cells expressing cmplx(ΔN)–GFP compared to cells stimulated with 100 µM ATP, respectively. In contrast, when cells expressing cmplx(ΔC)–EGFP were stimulated with UTP, halftimes of LTR fluorescence decay were not different from wild-type cells and cells expressing cmplx(wt)–GFP stimulated with ATP, but significantly shorter than in wild-type (*P* = 0.02) or cells expressing cmplx(ΔN)–GFP (*P* = 0.005) stimulated with UTP. This indicates, in line with a previous study (Dhara et al., 2014), that the C-terminus of complexin-2 clamps fusion pore expansion and that FACE-induced fusion pore expansion likely depends on inhibiting complexin-2 association with fused lamellar bodies. We next analysed the impact of Syt7 expression on complexin-2 translocation. Expression of Syt7(wt)–GFP did not affect inhibition of cmplx(wt)–GFP translocation and even resulted in a moderate acceleration of fusion pore expansion in cells stimulated with 100 µM ATP (Fig. 5C,D). However, in cells expressing Syt7(C_2_A*)–GFP, cmplx(wt)–GFP translocated to fused lamellar bodies in the presence of FACE. Halftimes of LTR fluorescence decay were also significantly increased by 59.0% (*P* = 0.04) and 116.4% (*P* = 0.02) when compared to wild-type cells or cells expressing Syt7(wt)–GFP, respectively. This effect was similar to the effect observed following fusions in the absence of FACE (Fig. 5C,D). In addition, the difference in LTR fluorescence decay between cells expressing Syt7(wt)–GFP and cells expressing Syt7(C_2_A*)–GFP was much more pronounced in cells overexpressing cmplx(wt)–GFP (63.6% versus 116.4%, respectively, see Fig. 4B and Fig. 5D). These data suggest that Ca^2+^ binding to the C_2_A domain of Syt7 antagonises complexin-2 recruitment to fused lamellar bodies and thereby facilitates accelerated fusion pore expansion.

### The C_2_A domain of Syt7 impairs Ca^2+^-dependent lamellar body exocytosis in ATII cells

**Fig. 6.**
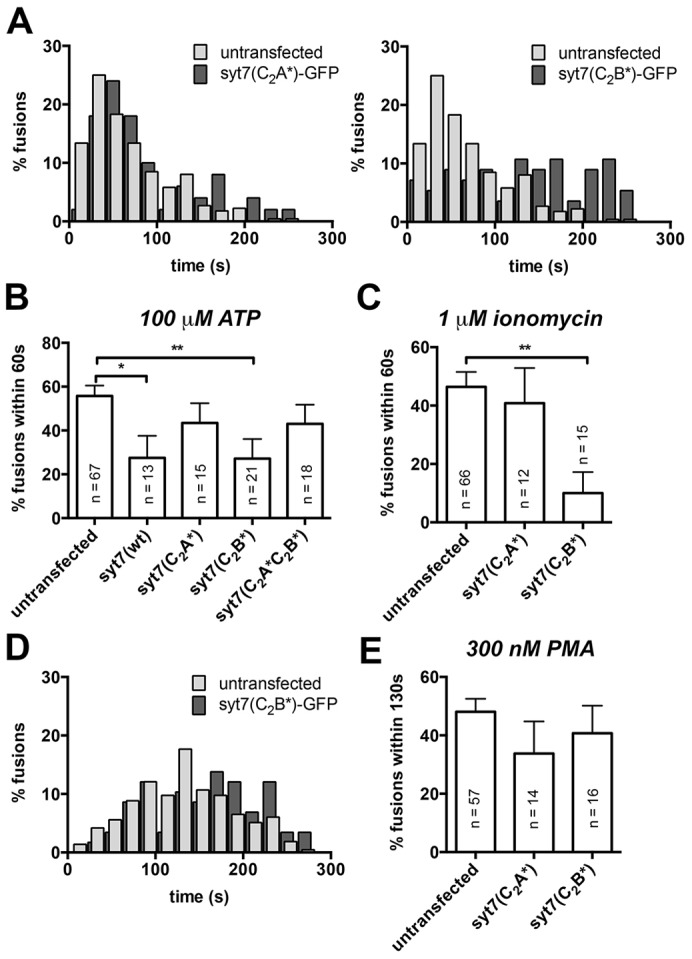
**The C_2_A domain of Syt7 impairs Ca^2+^-dependent lamellar body exocytosis in ATII cells.** (A) Expression of Syt7(C_2_B*)–GFP (right), but not Syt7(C_2_A*)–GFP (left) resulted in a significant left shift in the fusion delay histograms compared to untransfected cells following stimulation with 100 µM ATP. (B) The percentage of fusions occurring within 60 s of stimulation with 100 µM ATP (during the transient rise in intracellular Ca^2+^, see Fig. 4A) was significantly reduced in cells expressing Syt7(wt)–GFP or Syt7(C_2_B*)–GFP compared to untransfected cells. Transfection of ATII cells with Syt7 mutants with impaired Ca^2+^ binding to the C_2_A domain [Syt7(C_2_A*)–GFP or Syt7(C_2_A*C_2_B*)–GFP] did not impact on Ca^2+^-dependent fusion activity. (C) Following stimulation with 1 µM ionomycin, a Ca^2+^ ionophore that results in a strong long-lasting elevation of the cytoplasmic Ca^2+^, the effect of Syt7(C_2_B*)–GFP on inhibiting lamellar body exocytosis was even more significant than following stimulation with ATP. Expression of Syt7(C_2_A*)–GFP had no significant impact on fusion activity within 60 s of stimulation. (D) When lamellar body exocytosis was stimulated with 300 nM PMA, which does not result in any significant increase in the cytoplasmic Ca^2+^ concentration (supplementary material Fig. S2) expressing Syt7(C_2_B*)–GFP did not alter fusion kinetics. (E) No significant difference in the percentage of fusions occurring within 130 s of stimulation with PMA (when ∼50% of fusion occurred in untransfected control cells) were observed between untransfected cells and cells expressing either Syt7(C_2_A*)–GFP or Syt7(C_2_B*)–GFP. Results are mean±s.e.m. *P<0.05; **P<0.01.

We also analysed the impact of Syt7 expression on lamellar body exocytosis. In initial experiments we found that expression of Syt7(C_2_B*)–GFP resulted in a significant right shift in the fusion delay histograms compared to untransfected cells, indicating a shorter delay between stimulus and fusion (*P*<0.0001, median delay was 137 s and 50.6 s for Syt7(C_2_B*)–GFP-transfected and untransfected cells, respectively). This delay in lamellar body exocytosis upon stimulation with 100 µM ATP was not observed in cells transfected with Syt7(C_2_A*)–GFP (Fig. 6A). Initially, we assumed that these findings indicate a dependence of lamellar body exocytosis and fusion with the plasma membrane on the C_2_B domain of Syt7. However, when further analysing lamellar body fusion activity we found that also expression of Syt7(wt)–GFP caused a delay in lamellar body fusion with the plasma membrane upon stimulation (*P*<0.004, median delay was 86.2 s and 50.6 s for Syt7(wt)–GFP-transfected and untransfected cells, respectively). Moreover, the percentage of fusions occurring within 60 s of stimulation with 100 µM ATP (during the transient rise in intracellular Ca^2+^, see Fig. 4A) was significantly reduced in cells expressing Syt7(wt)–GFP (*P* = 0.02) or Syt7(C_2_B*)–GFP (*P* = 0.005) when compared to untransfected cells. However, transfection of ATII cells with Syt7 mutants with impaired Ca^2+^ binding to the C_2_A domain did not impact on fusion activity (Fig. 6B). These results rather suggest that the Syt7 C_2_A domain impairs fusion of lamellar bodies with the plasma membrane in the presence of Ca^2+^. To test this hypothesis, we stimulated lamellar body exocytosis using 1 µM ionomycin, a Ca^2+^ ionophore, which results in a strong long-lasting elevation of the cytoplasmic Ca^2+^ concentration (Frick et al., 2001). Under conditions of elevated Ca^2+^ concentrations, the effect of Syt7(C_2_B*)–GFP on inhibiting lamellar body exocytosis was even more significant than following stimulation with ATP (the percent of fusions occurring within 60 s of ionomycin treatment were reduced from 46.4±5.1% in untransfected cells to 10.0±7.2% in cells expressing Syt7(C_2_B*)-GFP, respectively, mean±s.e.m, *P* = 0.002). Expression of Syt7(C_2_A*)–GFP, by contrast, had no significant impact on fusion activity (*P* = 0.67) (Fig. 6C). Consistent with this, when lamellar body exocytosis was stimulated with 300 nM PMA, which does not result in any significant increase in the cytoplasmic Ca^2+^ concentration [see supplementary material Fig. S2 (Frick et al., 2001)], expressing Syt7(C_2_B*)–GFP did not change fusion kinetics (Fig. 6D). No significant difference in the percentage of fusions occurring within 130 s of stimulation with PMA (when ∼50% of fusion occurred in untransfected control cells) were observed between untransfected cells and cells expressing either Syt7(C_2_A*)–GFP or Syt7(C_2_B*)–GFP (Fig. 6E). Taken together these data strongly suggest that binding of Ca^2+^ to the C_2_A domain of Syt7 expressed on lamellar bodies hinders lamellar body exocytosis.

In summary, our results provide strong evidence that the C_2_A domain of Syt7 provides a link between FACE and fusion pore dilation during the post-fusion stage of lamellar body exocytosis in primary ATII cells. However, the C_2_A domain of Syt7 also impacts on fusion of lamellar bodies with the plasma membrane during the pre-fusion phase.

## DISCUSSION

Following lamellar body fusion with the plasma membrane, the vesicle cargo – pulmonary surfactant, a water insoluble, macromolecular complex of lipids and proteins – largely remains entrapped within the fused vesicles (Dietl and Haller, 2005). It has been demonstrated that fusion pores behave as mechanical barriers for release (Haller et al., 2001; Singer et al., 2003) and Ca^2+^-dependent fusion pore expansion is essential for efficient secretion of surfactant (Haller et al., 2001; Miklavc and Frick, 2011; Miklavc et al., 2012; Miklavc et al., 2011; Miklavc et al., 2013). Recently, we have demonstrated that FACE (the localised and transient Ca^2+^ entry at the site of vesicle fusion) provides the Ca^2+^ necessary for fusion pore expansion, even for late exocytic events (Miklavc and Frick, 2011). However, the molecular mechanisms demonstrating how FACE is actually translated into forces promoting fusion pore expansion were missing. Our present findings suggest that Syt7 provides a link between FACE, the local rise in Ca^2+^ and initial fusion pore expansion. This is in line with findings analysing fusion pore dynamics during exocytosis of large vesicles in non-neuronal cells, including insulin-secreting granules of β-pancreatic cells (Gao et al., 2000), large dense-core vesicles in PC12 cells (Zhang et al., 2010) and lysosomes (Jaiswal et al., 2004). In addition, the observed localisation of Syt7 on lamellar bodies is in line with previous reports that Syt7 is present on lysosomes and lysosome-related organelles (Jaiswal et al., 2002; Martinez et al., 2000; Reddy et al., 2001).

Exocytosis of lamellar bodies persists for up to 20 min after stimulation (Dietl et al., 2010) and a rise in cytoplasmic Ca^2+^ is the most potent trigger for lamellar body exocytosis (Haller et al., 1999) However, due to the slow kinetics of lamellar body exocytosis (Frick et al., 2001) and depending on the mode of stimulation, most fusions occur when Ca^2+^ levels have returned to baseline (∼120 nmol/l) (Dietl et al., 2012; Miklavc et al., 2014). Hence, the presence of a high-affinity Ca^2+^ sensor like Syt7 on lamellar bodies is ideally suited to link the transient and locally restricted Ca^2+^ signal induced by FACE to fusion pore dilation in newly fused vesicles.

Our data suggest a specific role for Ca^2+^ binding to the C_2_A domain of Syt7 that promotes fusion pore expansion of fused lamellar bodies. In addition, our data support a mechanism, whereby Ca^2+^ binding to the C_2_A domain results in inhibition of complexin-2 recruitment to the site of lamellar body fusion following fusion. This is in line with previous reports that suggest, that complexin binds to SNARE complexes (Südhof, 2013) leading to restriction of fusion pore dilation and that this mechanism is antagonised by synaptotagmins (Dhara et al., 2014). However, we cannot exclude that other mechanisms, apart from inhibition of complexin-2 translocation post fusion, also contribute to translating FACE and Ca^2+^ binding to Syt7 into forces promoting fusion pore expansion. The composition of the fusion pore, whether lipidic or protein-lined, still remains elusive (Chernomordik et al., 2006; Sørensen, 2009). It has been suggested that the transmembrane domains of SNARE proteins line the fusion pore (Han et al., 2004) and that interaction of synaptotagmins (Syt1) with SNARE proteins might drive lateral separation of the SNARE proteins to expand the pore (Lynch et al., 2008; Martens et al., 2007). These interactions are dependent on Ca^2+^ binding to C_2_ domains (Lynch et al., 2008). Similar dependency of fusion pore expansion on C_2_ domains was found in chromaffin cells from Syt7-knockout mice, albeit here an intact C_2_B domain supported full expansion of the fusion pore (Segovia et al., 2010). Further research is warranted to better understand the biophysical mechanism underlying synaptotagmin-driven fusion pore expansion in various systems. Alternatively, we cannot fully exclude that the impact of the mutation of C_2_A domain on Ca^2+^-dependent fusion pore expansion results from destabilisation of Syt7 (Maximov et al., 2008). In any case, expression of Syt7 and the availability of functional C_2_A domains is required for FACE-dependent fusion pore expansion.

In addition to modulating fusion pore kinetics, Syt7 could also play a role in stabilisation of the fusion pore (Jaiswal et al., 2004; Segovia et al., 2010). During lysosome exocytosis, Syt7 stabilises the fusion pore and prevents full fusion and collapse of the vesicle into the plasma membrane (Jaiswal et al., 2004). Such stabilisation of the fusion pore (i.e. controlled dilation and preventing full collapse or flattening of the fused lamellar body) could also be important for secretion of surfactant. Squeezing of surfactant through a narrow fusion pore (Haller et al., 2001; Miklavc et al., 2012) might be essential for proper transformation of surfactant from a lamellar into a more tubular structure to facilitate insertion into the surfactant layer lining the hypohase (Goerke, 1998). Alternatively, preventing rapid release of surfactant might aid in sequential or selective release of various lamellar body cargos (Tsuboi et al., 2004). However, additional experiments ablating Syt7 expression in primary ATII cells are required to test this hypothesis. This requires establishing of new techniques and is subject of on-going research including isolation of differentiated ATII cells from mice or maintaining ATII cell differentiation *in vitro* for extended periods for knockout approaches. Efficient knockdown of transmembrane proteins has not yet been achieved on the protein level in isolated primary ATII cells [mainly as a result of the short time-window for maintaining differentiated cells and due to the slow membrane turnover in these cells in cell culture conditions (Albrecht et al., 2010)].

Mice lacking Syt7 are viable, fertile and do not display any significant abnormalities or respiratory phenotypes (Chakrabarti et al., 2003; Maximov et al., 2008). Despite the fact that efficient secretion of pulmonary surfactant is vital for lung function (Dietl et al., 2004), several explanations could account for the missing phenotype in mice lacking Syt7 or Syt7 C_2_A domains. First of all, it still needs to be determined whether FACE and FACE-dependent fusion pore expansion is also present in murine ATII cells. So far this has been neglected owing to the lack of established procedures to obtain sufficient functional primary ATII cells from mice. Moreover, all assays to study lamellar body exocytosis and surfactant secretion are well established for primary ATII cells from rat (Haller et al., 2001; Haller et al., 1998), as rat, but not mouse ATII cells, resemble those of humans (Mair et al., 2004). Second, it has been shown that, next to fusion pore expansion actomyosin-dependent compression of fused lamellar bodies is essential for active expulsion of surfactant (Miklavc et al., 2012; Miklavc et al., 2009). Such force-generating mechanisms could potentially compensate for incomplete or delayed fusion pore dilation. Third, it is well established that surfactant secretion can be adjusted by increasing the number of lamellar bodies fusing with the plasma membrane and/or changes in surfactant loading into lamellar bodies (Dietl et al., 2004; Dietl et al., 2001; Haller et al., 1999). This could actually be relevant in Syt7-knockout mice as our data suggest that Syt7 (the C_2_A domain) impairs Ca^2+^-induced lamellar body exocytosis. None of these mechanisms has been investigated in mice lacking Syt7. Additionally, it cannot be excluded that compensatory expression of synaptotagmin isoforms is induced to maintain this vital function.

Our observation that (over)expression of the Syt7 C_2_A domain impairs Ca^2+^-induced lamellar body exocytosis is in contrast to most previous reports, where Ca^2+^ binding to the C_2_A domains has been found as trigger for exocytosis, although individual observations also suggest that, depending on the mode of stimulation, Syt7 can act as inhibitor of lysosome exocytosis (Jaiswal et al., 2004). Whether Ca^2+^ binding to the C_2_A domain of Syt7 expressed on lamellar bodies hinders lamellar body exocytosis by impeding docking of lamellar bodies to the plasma membrane or directly impacts on the fusion of lamellar bodies with the plasma membrane remains to be answered. Although we cannot fully explain our observation, one possibility is that, in line with our results for fusion pore expansion, excess Syt7 and, in particular, Ca^2+^ binding to the C_2_A domain, prevents complexin-2 binding to SNARE complexes during the pre-fusion phase. Complexin binding to SNAREs has been demonstrated to activate the SNARE–SM-protein complex (Maximov et al., 2009) and that at least part of complexin competes with synaptotagmin for SNARE complex binding (Südhof, 2013). Alternatively, it is possible that proteins other than Syt7 constitute the Ca^2+^ sensor for lamellar body fusion with the plasma membrane and that excess Syt7 C_2_A interferes with the Ca^2+^ sensor. Annexin II has been found as a Ca^2+^ sensor in lamellar body exocytosis and mediates membrane fusion through its interaction with SNARE proteins (Chattopadhyay et al., 2003; Wang et al., 2007). However, further experiments are required to better understand the role of Syt7 during the pre-fusion phase of lamellar body exocytosis.

Overall, our study demonstrates that Syt7 is expressed on lamellar bodies and suggests that Syt7 provides a molecular link between FACE and fusion pore dilation. Specifically, binding of Ca^2+^ to the C_2_A domain of Syt7 facilitates fusion pore dilation during the exocytic post-fusion phase. This possibly involves inhibition of complexin-2 translocation to lamellar bodies after fusion. These findings add to the picture that Syt7 modulates fusion pore expansion in large secretory organelles and extend our picture that lamellar bodies themselves harbour the molecular inventory to facilitate secretion of pulmonary surfactant during the exocytic post-fusion phase. Moreover, our results suggest that tight regulation of Syt7 levels on lamellar bodies (and probably a balanced interplay between Syt7 and complexin-2) is essential in order that lamellar body exocytosis is not disrupted during the exocytic pre-fusion phase.

## MATERIALS AND METHODS

### Materials

Anti-synaptotagmin7 poly-clonal antibody (pAb) and anti-complexin-2 antibodies were purchased from Synaptic Systems (Göttingen, Germany), anti-P180 lamellar body protein (ABCa3) monoclonal antibody (mAb) was purchased from Abcam (Cambridge, UK). Fluorescently labelled secondary antibodies were obtained from Molecular Probes (Life Technologies, Karlsruhe, Germany).

### Cell isolation

ATII cells were isolated from Sprague-Dawley rats according to the procedure of Dobbs et al. (Dobbs et al., 1986) with minor modifications as recently described (Miklavc et al., 2010). After isolation, cells were seeded on glass coverslips, cultured in MucilAir (Epithelix, Switzerland), and used for experiments for up to 48 h after isolation, before de-differentiation affects lamellar body exocytosis. All animal experiments were performed according to approved guidelines.

### Experimental conditions

Experiments were performed as recently described (Miklavc et al., 2010). For all experiments, cells were kept in bath solution (in mM: 140 NaCl, 5 KCl, 1 MgCl_2_, 2 CaCl_2_, 5 glucose, 10 Hepes pH 7.4). To efficiently induce lamellar body fusions ATII cells were treated with various known and potent agonists for lamellar body fusion and surfactant secretion: ATP (100 µM), UTP (100 µM), phorbol 12-myristate 13-acetate (PMA; 300 nM) or ionomycin (1 µM) (all from Sigma, Schnelldorf, Germany). Concentrations were chosen to induce maximum fusion response. For LTR diffusion experiments cells were preloaded for 15 min with 200 nM of either dye in MucilAir, washed twice in bath solution and kept in bath solution for the duration of the experiment. All fluorescent dyes were purchased from Molecular Probes (Life Technologies, Karlsruhe, Germany).

### Plasmids

Wild-type cDNA encoding rat Syt7 was a generous gift from Thomas C. Südhof (University of Stanford, CA, USA). Mutations in the C_2_A and C_2_B Ca^2+^-binding domains (see Fig. 2A) were introduced using the QuikChange Lightning multi site-directed mutagenesis kit (Agilent Technologies, Waldbronn, Germany). The following primers were used to replace D for A in the C_2_A domain: D225A_D227A (sense: 5′-CTCCAGGTCCTGGCTTATGCCCGTTTCAGCCGC-3′) and D233A (sense: 5′-GTTTCAGCCGCAATGCCGCCATTGGGGAG-3′). The following primers were used to replace D for A in the C_2_B domain: D303A (sense: 5′-CGGGGGCACATCAGCCCCCTATGTGAAGG-3′) and D357A_D359A (sense: 5′-CATCATCACTGTCATGGCCAAAGCCAAGCTCAGCCGCAATG-3′). Mutations were confirmed by sequencing (GATC Biotech, Konstanz, Germany). Wild-type and mutant Syt7 was cloned into pEGFP-N1 (Clontech, USA) using the In-Fusion HD cloning kit (Clontech, USA) and using primers to insert a 5× glycine linker between Syt7 and GFP.

cDNA encoding wild-type Syt1 was amplified from brain lysate and cDNA encoding Syt4 was from Jane Sullivan (University of Washington, Seattle, WA; originally from Addgene, plasmid 12503). Similar to Syt7, Syt1 and Syt4 were cloned into pEGFP-N1 using primers to insert 5× glycine linkers between Syt1 or Syt4 and GFP, respectively. cDNA encoding complexin-2 was amplified from ATII cell lysate and cloned into pEGFP-N1 as recently described (An et al., 2010) to yield cmplx(wt)–GFP (full length), cmplx(ΔN)–GFP (amino acids 28–134) and cmplx(ΔC)–GFP (amino acids 1–72).

All constructs were confirmed by DNA sequence analysis (GATC Biotech, Konstanz, Germany).

### Semi-quantitative RT-PCR

Total RNA was isolated from 10^6^ ATII cells 24 h after isolation using RNeasy MiniKit (Qiagen, Hilden, Germany). Reverse transcription was performed on 0.8 µg to 1.3 µg total RNA using the SuperScript VILO cDNA synthesis kit according to manufacturer's protocol. The following validated QuantiTect primer assays (Qiagen, Hilden Germany) were used: HMBS, Rn_Hmbs_1_SG; Syt1, Rn_Syt1_2_SG; Syt-2, Rn_Syt2_1_SG; Syt3, Rn_Syt3_1_SG; Syt5, Rn_Syt5_1_SG; Syt6, Rn_Syt6_1_SG; Syt7, Rn_Syt7_1_SG; Syt-9, Rn_RGD:621169_1_SG; Syt10, Rn_Syt10_2_SG; complexin-1, Rn_Cpl1_1_SG; complexin-2, Rn_Cpl2_1_SG; complexin-3, Rn_Cpl3_1_SG; complexin-4, Rn_Cpl4_1_SG. Amplification was performed on a realplex2 mastercycler (Eppendorf, Hamburg, Germany) using the XPress Syber Green ER qRT-PCR super mix. Each reaction was carried out on cDNA from three or more independent isolations (cDNAs were used at 1-, 10- and 100-fold dilutions). Specificity of PCR reactions was confirmed by melting points analysis of PCR products. Realplex software (Eppendorf, Hamburg, Germany) was used for data acquisition and analysis. Correction for PCR performance as well as quantification relative to housekeeping gene HMBS was carried out as described (Pfaffl, 2001).

### Western blotting

ATII cells were washed twice in PBS, solubilised in lysis buffer, separated by SDS-PAGE and transferred onto nitrocellulose. Ponceau staining of blots was performed before immuno-labelling to normalise for sample loading. Immuno-detection of Syt7 was performed using anti-Syt7 pAb (1∶500) and chromogenic detection of alkaline-phosphatase-labelled secondary antibody (WesternBreeze anti-rabbit, Invitrogen, Karlsruhe, Germany). The specificity of anti-Syt7 pAb was validated by competition with 10 µg/ml control protein (amino acids 46–133 of rat synaptotagmin 7, Synaptic Systems, Göttingen, Germany) (supplementary material Fig. S4).

### Immunofluorescence

For immunofluorescence staining, cells were washed twice in DPBS (pH 7.4, Biochrom, Berlin, Germany) fixed for 20 min in 4% paraformaldehyde (Sigma, Schnelldorf, Germany) in DPBS, and permeabilised for 10 min with 0.2% saponin and 10% FBS (Thermo Scientific, Bonn, Germany) in DPBS. Cells were subsequently stained with primary (1∶100) and secondary (1∶400) antibodies in PBS, 0.2% saponin and 10% FBS. Images were taken on an inverted confocal microscope (Leica TCS SP5, Leica, Germany) using a 63× lens (Leica HCX PL APO lambda blue 63.0× 1.40 NA oil UV lens). Images for the blue (DAPI), green (Alexa Fluor 488) and red (Alexa Fluor 568) channels were taken in sequential mode using appropriate excitation and emission settings.

### Live-cell fluorescence imaging

For Lysotracker Red (LTR, LifeTechnologies, Germany) diffusion experiments, cells were incubated with LTR for (100 nM) for 10 min, washed twice in PBS and mounted in bath solution before start of the experiment. LysoTracker dyes accumulate in lamellar bodies and rapidly diffuse out of the vesicle after fusion (Haller et al., 1998; Haller et al., 2001). Experiments were performed on the iMic digital microscope (Till Photonics, Germany) with a 488 nm excitation filter for actin GFP and 568 nm excitation filter for LTR. FM1-43 experiments were performed on an iMic digital microscope (Till Photonics, Germany) or on a Cell Observer inverse microscope (Zeiss, Germany). For FM1-43 and combined fura-2 and FM1-43 experiments cells were illuminated for 50 ms at a rate of 0.3–0.5 Hz at each excitation wavelength (340 and 380 nm for fura-2; 480 nm for FM 1-43). A 495 nm (Observer) and a 520 nm dichroic mirror (iMic) were used to deflect excitation light. In this setting, channel crosstalk between the FM 1-43 fluorescence and the fura-2 ratio would lead to small under-estimations of [Ca^2+^]_c_ as described in detail earlier (Haller et al., 1998).Images were acquired using MetaFluor (Molecular Devices, Ismaning, Germany) or iMic Online Analysis (Till Photonics, Germany). Similar levels of Syt7(wt)–GFP, Syt7(C_2_A*)–GFP, Syt7(C_2_B*)–GFP, Syt7(C_2_A*C_2_B*)–GFP were expressed in primary ATII cells for functional studies analysing fusion pore opening (supplementary material Fig. S4).

### Image analysis and data presentation

Images were analysed using MetaFluor Analyst (Molecular Devices, Ismaning, Germany) and iMic Offline analysis software (Till Photonics, Germany) and ImageJ (NIH, Bethesda, USA) as recently described (Miklavc et al., 2011).

Diffusion of LTR out of and FM1-43 into newly fused lamellar bodies was analysed as a direct means to compare fusion pore expansion following lamellar body fusion under various conditions. To compare kinetics of LysoTracker diffusion we analysed half-times of fluorescence decay following lamellar body fusion. LysoTracker fluorescence was normalized to that of non-fusing vesicles to compensate for bleaching and fitted to an one-phase decay. In all experiments, the size and shape of lamellar bodies analysed was similar, therefore we can exclude size effects impacting on changes in vesicle fluorescence (supplementary material Fig. S4). Owing to the slow kinetics of FM1-43 labelling of fused vesicles, we analysed the initial slope (15 s after fusion) of the increase of FM1-43 fluorescence.

To eliminate potential interference with fura-2 ratio calculations in combined fura-2 and FM1-43 experiments, ura-2 fluorescence was determined in ring-like (which are peri-vesicular) regions of interest (800–1000 nm wide) surrounding the fused FM-1-43-stained lamellar body. The onset of the Ca^2+^ rise was specified as the time-point when the increase in the fura-2 ratio exceeded its s.d. by twofold. The peak amplitude for fura-2 signals was specified as the difference between fura-2 ratio values of the last time-point before onset of FACE and the maximum value within 10 s thereof. For analysis of cmplx(wt)–GFP and cmplx(ΔC)–GFP recruitment to fused lamellar bodies, mean GFP fluorescence was analysed in a peri-vesicular region of interest surrounding individual lamellar bodies. Fluorescence was normalised to the fluorescence at onset of lamellar body fusion as indicated by loss of LTR from the vesicle.

MS Excel and GraphPad Prism 5 were used for statistics, curve fitting and graph design. Unless otherwise stated, all data are presented as mean±s.e.m.

### Calculation of cytoplasmic Ca^2+^ concentration

We estimated the intracellular free Ca^2+^ concentration in isolated ATII cells as described in detail previously (Grynkiewicz et al., 1985; Haller et al., 1999; Haller et al., 1996) using the equation: [Ca^2+^]_c_ = *K*_d_×[(*R*−*R*_min_)/(*R*_max_−*R*)]×(*S*_f2_/*S*_b2_), (see below for explanation of the equation terms).

ATII cells were seeded in perfusion chambers (Ibidi, Martinsried, Germany) and stained with fura-2 AM (3 µM for 20 min). The ratio (*R*) was calculated from the 340 and 380 nm excitation intensities in unstimulated cells in normal experimental bath solution. *R*_min_ was measured after Ca^2+^ ionophore ionomycin (20 µM) was added to a Ca^2+^-free perfusion solution (in mM: 135 NaCl, 5 KCl, 1 MgCl_2_, 5 glucose, 5 Hepes pH 7.4, 5 EGTA). *R*_max_ was measured after ionomycin (20 µM) addition to Ca^2+^-containing perfusion solution (in mM: 134 NaCl, 5 KCl, 1 MgCl_2_, 5 CaCl_2_, 5 glucose, 5 Hepes pH 7.4). The proportionality coefficient *S*_f2_ was measured as the maximal 380 nm fluorescence intensity after ionomycin addition to Ca^2+^-free solution. The second proportionality coefficient, S_b2_, was measured as the minimal 380 nm fluorescence intensity after ionomycin addition to Ca^2+^-containing solution. We corrected both coefficients for the background fluorescence which was measured after MnCl_2_ quench (in mM: 140 NaCl, 5 Hepes pH 7.4, 5 MnCl_2_) at the end of the experiment. We used 224 nM as a *K*_d_ value for fura-2 (Grynkiewicz et al., 1985).

## Supplementary Material

Supplementary Material
