# Supplementary material for: Synaptotagmin-7 links fusion-activated Ca2+ entry and fusion pore dilation
Source: J Cell Sci. 2014 Dec 15;127(24):5218–27. doi: 10.1242/jcs.153742 (PMC4265738; doi:10.1242/jcs.153742)
Supplement: Supplementary Material [file supp_127_24_5218__index.html]

Synaptotagmin-7 links fusion-activated Ca2+ entry and fusion pore dilation — Supplementary Material 

# Synaptotagmin-7 links fusion-activated Ca2+ entry and fusion pore dilation

## JCS153742 Supplementary Material

**Files in this Data Supplement:**

- **Supplementary Material**
