# Supplementary material for: Synaptotagmin-7 links fusion-activated Ca2+ entry and fusion pore dilation
Source: J Cell Sci. 2014 Dec 15;127(24):5218–27. doi: 10.1242/jcs.153742 (PMC4265738; doi:10.1242/jcs.153742)

Supplement Information

Suppl. Figure 1

(A) Syt1(wt)-GFP (left) and syt4(wt)-GFP (right) also localise on LB membranes as confirmed by perivesicular localisation on LTR positive LBs. Scale bar: 3 μm

(B) Expression of syt1(wt)-GFP (left) or syt4(wt)-GFP (right) does not impact on LTR fluorescence decay and hence fusion pore expansion, respectively.

Suppl. Figure 2

In contrast to cells stimulated with 100 μM ATP (Fig. 4B) no difference in LTR fluorescence decay between cells expressing syt7(wt)-GFP, syt7(C_2A*)-GFP and syt7(C_2B*)-GFP was observed in cells stimulated with either 100 μM UTP (A) or 300 nM PMA (B), conditions where FACE is not activated (Miklavc et al., 2011).

(C) Response of ATII cells to different modes of stimulations. Ca^{2+}-responses following stimulation of ATII cells with either 100 μM ATP or 300nM PMA (stimulation was applied at t = 0s). Mean traces have been derived from 23 (ATP) and 11 (PMA) cells, respectively.

Suppl. Figure 3

(A) Real-time RT-PCR analysis of complexin transcripts in freshly isolated ATII cells from rat. Data are expressed as fold expression of housekeeping gene HMBS. Values are means from 3 individual cell isolations and are represented as means + SEM.

(B) complexin-2 (red) is localised in the cytoplasm of ATII cells as detected by indirect immunofluorescence. LBs were identified by staining with P180 lamellar body protein (green, ABCa3). Scale bar: 20 μm

Suppl. Figure 4

(A) Validating specificity of anti-syt7 antibody. Equal amounts of cell lysate from primary ATII cells were loaded on a gel (as confirmed by Ponceau staining, left) and Western Blot (right) was performed in the absence or presence of 10 μg / ml control protein (aa 46 - 133 of rat synaptotagmin-7). Presence of recombinant syt-7 abolished the strong signal at ~40 kDa, indicating that the antibody specifically recognizes syt-7 expressed in primary ATII cells (light bands at other molecular weights are not affected by the competition with recombinant rat synaptotagmin 7).

(B) Expression levels of syt7(wt)-GFP, syt7(C2A*)-GFP, syt7(C2B*)-GFP, syt7(C2A*C2B*)-GFP were similar in cells used for functional studies. Mean syt7-GFP fluorescence was analysed in cells used for determining the impact of Ca^{2+}-binding to C2 domains of syt-7 (Fig. 4B). For quantitative comparison, cells from the same preparations were transfected in parallel with either syt7(wt)-GFP, syt7(C2A*)-GFP, syt7(C2B*)-GFP or syt7(C2A*C2B*)-GFP, images were recorded at identical microscope settings and fluorescence intensities for individual cells were derived by measuring the mean fluorescence of the cell and subtracting the mean fluorescence of an adjacent area next to the cell to correct for background signal.

(C) Average LB sizes were similar for all experiments where LTR half-times were analysed. We can therefore exclude that differences in LB size effect the kinetics of the observed changes in vesicle fluorescence decay following LB fusion and fusion pore opening. Vesicle sizes are expressed as mean vesicle diameters, number of vesicles analysed = 55 for each condition.

Suppl. Figure 1

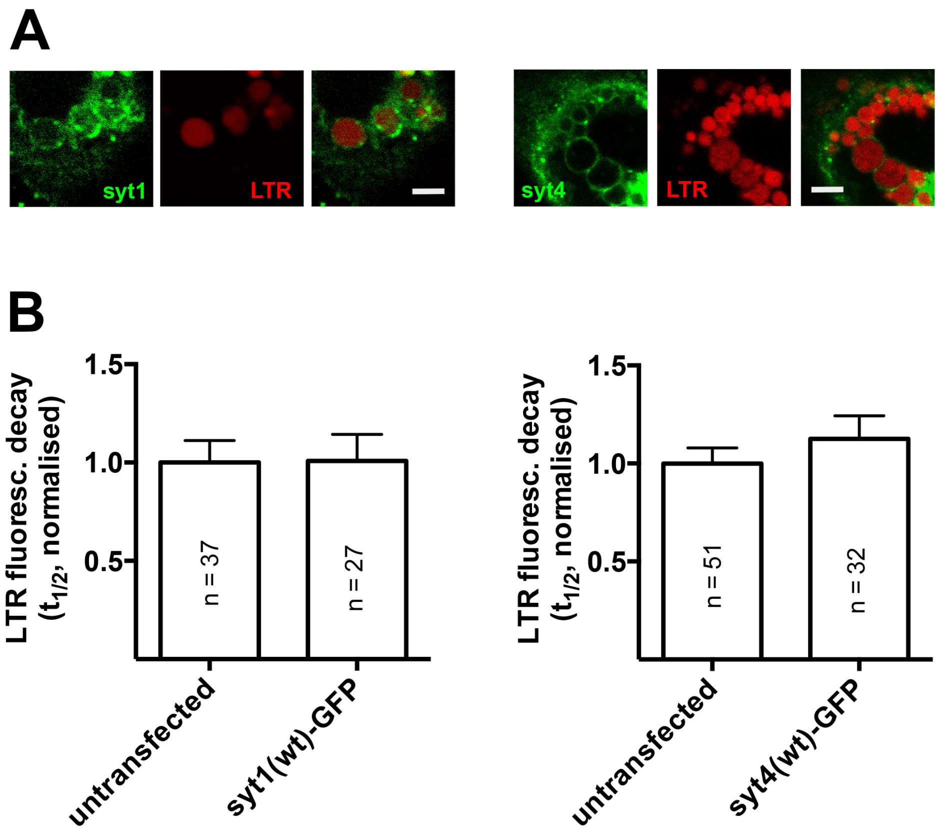

Suppl. Figure 2

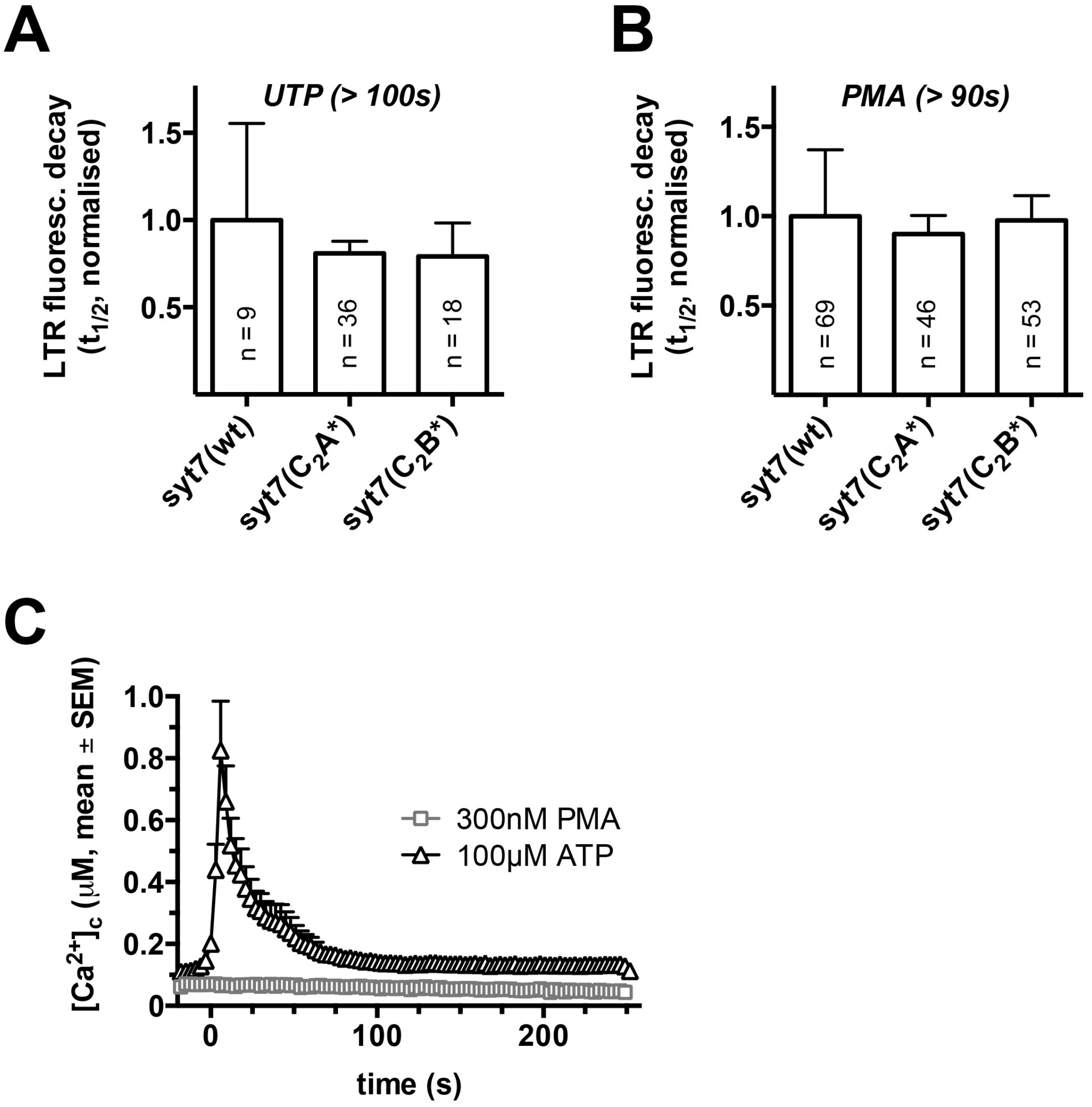

Suppl. Figure 3

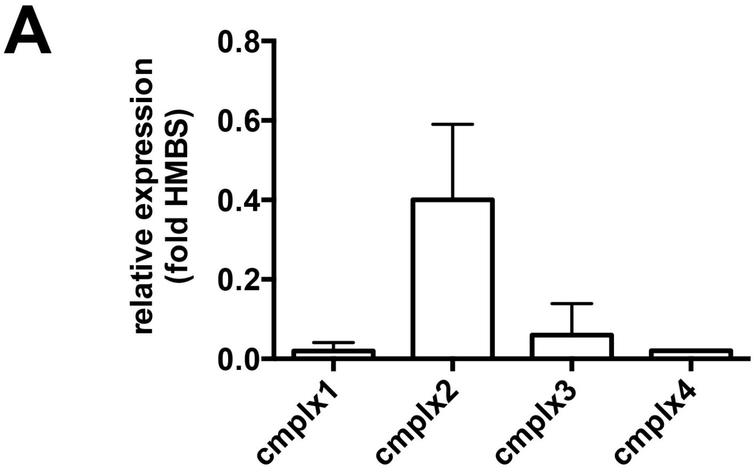

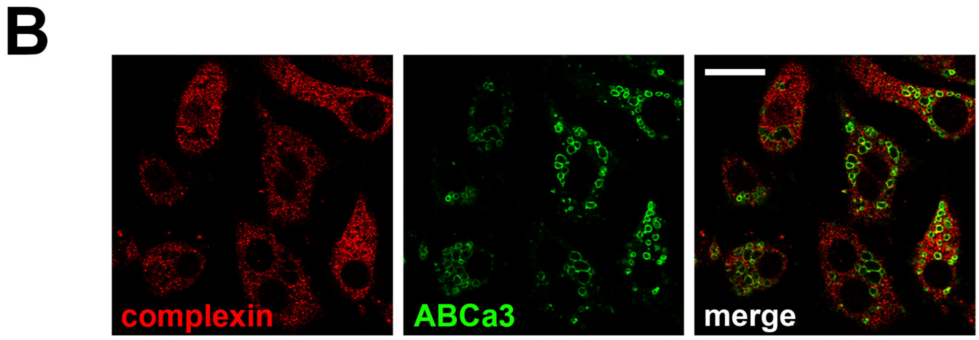

Suppl. Figure 4

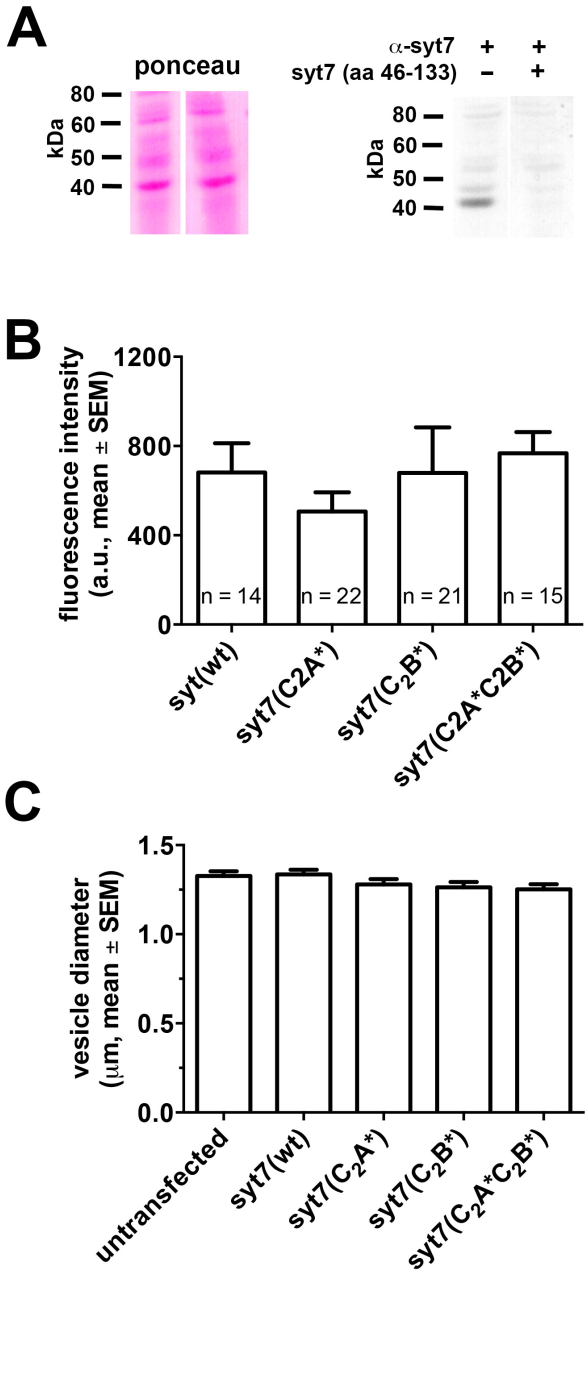

Supplement: Supplementary Material [file supp_127.24.5218_JCS153742.pdf]
